# Protocol for establishing a murine Toscana virus infection model and downstream cellular analysis of skin tissue and cells

**DOI:** 10.1016/j.xpro.2026.104885

**Published:** 2026-10-08

**Authors:** Yonca Keskek Turk, Ailish McCafferty-Brown, Clive S. McKimmie

**Affiliations:** 1 School of Medicine, University of Leeds, Leeds, UK; 2 Virus Host Interaction Team, Skin Research Centre, York Biomedical Research Institute, Hull York Medical School, University of York, York, UK; 3 Department of Medical Microbiology, Virology Unit, Faculty of Medicine, Hacettepe University, Ankara, Türkiye; 4 Institute for Regeneration and Repair, University of Edinburgh, Edinburgh, UK

**Keywords:** Flow Cytometry, Immunology, Microbiology, Model Organisms

## Abstract

Here, we present an *in vivo* approach to study virus-vector-host interactions during Toscana virus (TOSV) infection in mouse skin. We describe preparation of viral stocks and subcutaneous TOSV inoculation into dorsal upper foot skin, either alone, with sand-fly salivary gland extract (SGE), or following sand-fly biting. We then detail tissue collection and generation of single-cell suspensions. Finally, we describe flow cytometric detection of infected cells, magnetic isolation of dermal fibroblasts, and RNA extraction for transcriptomic analysis.

For complete details on the use and execution of this protocol, please refer to Keskek Turk et al.^1^

## Highlights

• Steps for subcutaneous Toscana virus inoculation into mouse upper foot skin
• Protocol for controlled sand fly blood-feeding prior to virus inoculation
• Enzymatic digestion of skin to generate viable single-cell suspensions
• Detection of infected skin cells by flow cytometry using an mCherry reporter virus

## Abstract (editor highlights)

Publisher’s note: Undertaking any experimental protocol requires adherence to local institutional guidelines for laboratory safety and ethics.

## Graphical abstract

**Figure:**
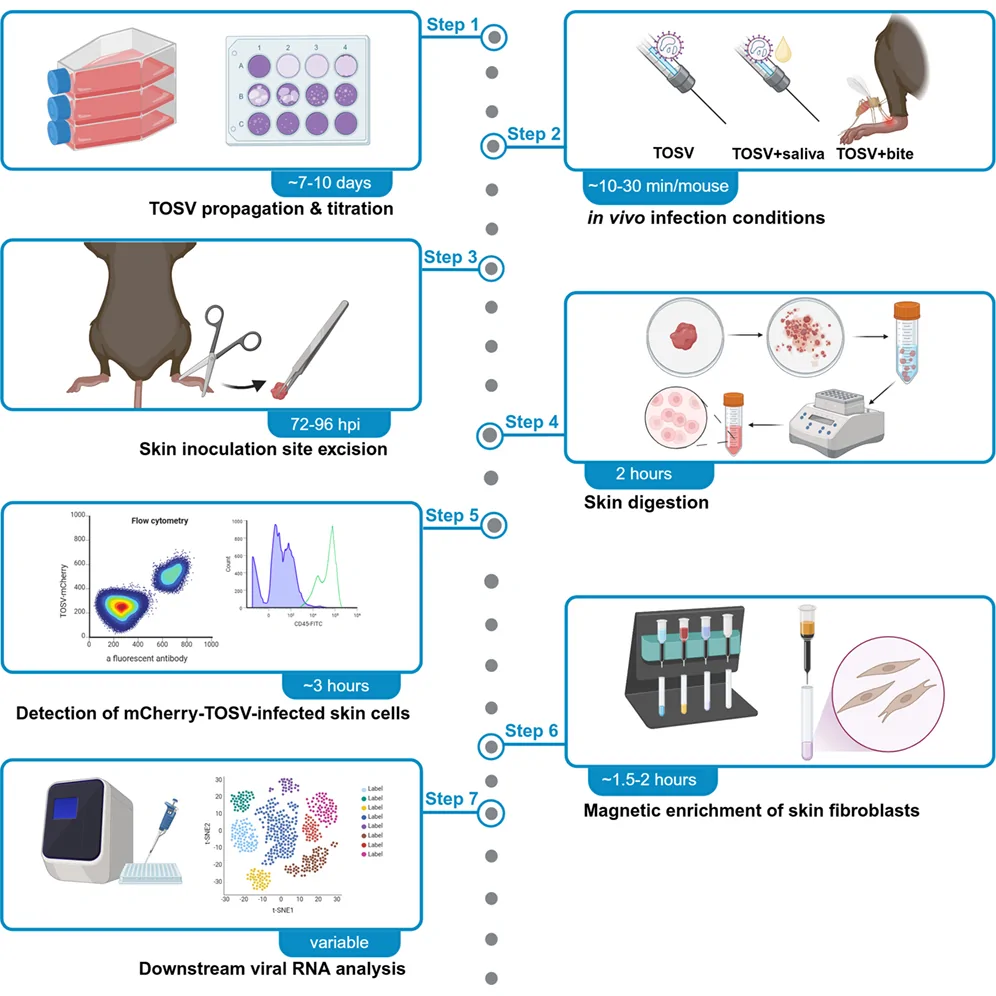

## Before you begin

Toscana virus (TOSV) is a sand fly-transmitted *phlebovirus* and the leading cause of viral neurological disease in the Mediterranean region,^2^ yet the impact of vector-derived salivary factors on the identity and susceptibility of skin-resident target cells during infection remains poorly defined.^1^

Here, we describe a protocol for establishing a murine skin infection model of TOSV that enables downstream cellular and molecular analyses of infected skin tissue and enables researchers to study vector-derived modulation of viral infection. This workflow combines subcutaneous infection of *Ifnar1*^−/−^ mice with TOSV inoculum, either prepared in combination with sand fly salivary gland extract (SGE) pre-injection or inoculated after an *in vivo* sand fly bite, followed by enzymatic digestion of skin and single-cell-based readouts. Detection of infected cells by flow cytometry is achieved using a fluorescent reporter virus (mCherry-TOSV). Optional steps include magnetic enrichment of fibroblasts and RNA extraction from whole skin tissue or enriched cell populations for downstream applications such as RT-qPCR or RNA sequencing. All procedures involving live viruses and animals must be performed in accordance with institutional biosafety and ethical regulations.

### Innovation

Existing protocols for studying arbovirus infection in mouse skin typically rely on needle inoculation of virus alone, without incorporating vector-derived salivary factors that are co-deposited during natural transmission. This omission is significant because vector saliva profoundly alters the skin microenvironment and can determine infection outcome.^1^ Furthermore, most published approaches assess infection through bulk tissue viral load measurements, without resolving the identity or differentiation state of infected cell populations in situ.

This protocol addresses these gaps through several key advances. First, it establishes a standardised workflow for co-delivering TOSV with sand fly SGE or into sand fly-bitten skin using custom 33-gauge microneedles and a Hamilton microsyringe system, enabling precise subcutaneous inoculation at the anatomical site of natural vector feeding. Second, it incorporates a recombinant mCherry-expressing TOSV^3^ into a multi-parameter flow cytometry pipeline that permits simultaneous detection of infected cells and phenotypic classification across immune and stromal lineages, including fibroblast subpopulations defined by markers such as Sca-1, CD90.2 and podoplanin. Third, it provides an optimised two-step MACS enrichment strategy (negative depletion followed by positive CD90.2 selection) that yields fibroblast populations of sufficient purity and RNA quality for downstream transcriptomic applications, including RNA sequencing from low-input skin samples.

Finally, by integrating these components into a single end-to-end workflow, this protocol enables researchers to move beyond bulk tissue readouts and interrogate how vector-derived factors reprogram specific skin-resident cell types to alter susceptibility to arbovirus infection.

### Institutional permissions

All animal procedures described in this protocol were approved by the University of Leeds and University of York Animal Welfare and Ethical Review Bodies (AWERB) and performed under UK Home Office Project Licence PP0258562, in accordance with the Animals (Scientific Procedures) Act 1986. Work with live TOSV and recombinant mCherry-TOSV was conducted under appropriate biosafety containment conditions as determined by institutional biological safety committees.

Researchers wishing to implement this protocol must obtain equivalent ethical approval for animal experimentation and biosafety authorisation for handling live arboviruses from their own institutions and national regulatory bodies before commencing work. Requirements will vary by jurisdiction and may include specific containment level designations for sand fly colony maintenance and arbovirus manipulation.

### Preparations

#### Preparation of mice and experimental grouping

**Timing: Variable (completed before experiment start)**

1. Assign age and sex matched *Ifnar1*^−/−^ mice (6–10 weeks of age) to experimental groups (e.g., TOSV alone, TOSV+SGE or infection into sand fly-bitten skin).

***Note:*** This protocol is not restricted to a specific sex. Mice were assigned according to colony availability in accordance with the 3Rs principles and each experiment was performed using internally age and sex matched cohorts. Fixed-timepoint and clinical sign experiments were conducted independently using separate matched cohorts.

2. Define virus titre, final injection volume (typically just 1-2 μL to better mimic small volumes deposited by biting insects) and tissue collection time points prior to beginning the experiment.
3. Ensure all procedures are approved by the relevant animal ethics committee and that personnel are trained in animal handling, inhalation anesthesia and subcutaneous injections.

***Note:*** This protocol was optimized using final inoculum volumes of 1-2 μL. The small anatomical area of dorsal upper foot skin limits the volume that can be delivered reproducibly by subcutaneous injection; therefore, larger volumes should be optimized before use.

#### Preparation of infection and tissue collection materials

**Timing: 1 h**

4. Prepare Hamilton 75 RN syringes (5 μL) fitted with custom 33G microneedles for subcutaneous injections.
5. Sterilize and arrange surgical scissors and forceps for tissue dissection.
6. Pre-label sterile 1.5 mL tubes for each mouse and tissue type.
7. Prepare collection tubes containing complete media or RNA stabilizer solution, as required.
8. Prepare ice buckets for temporary sample storage.

#### Preparation of reagents for downstream tissue processing and cell analysis (if applicable)

**Timing: ∼2 h**

9. Prepare tissue digestion solution containing Hank’s Balanced Salt Solution (HBSS), Collagenase P (1 mg/ml final), Dispase II (0.5 mg/mL final) and DNase I (0.1 mg/mL final).
10. Prepare and pre-cool: FACS buffer (PBS without Ca^2+^/Mg^2+^, 0.5% Fetal Calf Serum, 2 mM EDTA), MACS buffer (PBS without Ca^2+^/Mg^2+^, 0.5% Fetal Calf Serum, 2 mM EDTA) and RNA lysis buffers appropriate for downstream extraction kits.
11. Prepare antibody master mixes according to the planned flow cytometry panel.
12. Prepare Fc receptor blocking reagent and Live/Dead staining dye.

***Note:*** Prepare digestion solution volumes based on total sample number plus one extra reaction.

**CRITICAL:** Protect fluorescent dyes from light at all stages. Do not place Live/Dead dye mixture on ice, as DMSO containing solutions may precipitate.

## Key resources table

**Table:** 

REAGENT or RESOURCE	SOURCE	IDENTIFIER
**Antibodies**		

FITC-CD45 (1:200)	BioLegend	30-F11; Cat#103107; RRID: AB_312972
APC-CD11b (1:200)	BioLegend	M1/70; Cat#101211; RRID: AB_312795
APC-Cy7-MERTK (Mer) (1:200)	BioLegend	2B10C42; Cat#151519; RRID: AB_2876507
Brilliant Violet 421-Ly-6G (1:200)	BioLegend	1A8; Cat#127627; RRID: AB_2562567
PE-Ly-6C (1:200)	BioLegend	HK1.4; Cat#128007; RRID: AB_1186133
APC-MHC II (1:200)	BioLegend	M5/114.15.2; Cat#107613; RRID: AB_313328
Pe-Cy7-CD11c (1:200)	BioLegend	N418; Cat#117317; RRID: AB_493569
APC-Vimentin (1:200)	Biotechne	280618; Cat#IC2105A; RRID: AB_3654983
FITC-CD326 (Ep-CAM) (1:200)	BioLegend	G8.8; Cat#118208; RRID: AB_1134107
PE-CD31 (1:200)	BioLegend	390; Cat#102407; RRID: AB_312903
Pacific Blue-CD90.2 (Thy1.2) (1:200)	BioLegend	30-H12; Cat#105323; RRID: AB_1877204
Alexa Fluor 647-Podoplanin (1:200)	BioLegend	PMab-1; Cat#156203; RRID: AB_2750403
PE/Cy7-CD140a (PDGFR-α) (1:200)	BioLegend	APA5; Cat#135911; RRID: AB_2715973
PE-Ly-6A/E (Sca-1) (1:200)	BioLegend	W18174A; Cat#160905; RRID: AB_2910334
Zombie UV (1:50)	BioLegend	RUO; Cat#423107

**Bacterial and virus strains**		

Toscana Virus; strain 1812	Isolated from patient (Cusi et al^4^)	N/A
Toscana Virus; strain 1500590; lineage A	Alexander et al.^3^	N/A

**Biological samples**		

*Ifnar1*^−/−^ mice skin, spleen, lymph node, brain and foot tissues, and blood serum	(Keskek Turk et al.^1^)	N/A

**Chemicals, peptides, and recombinant proteins**		

Streptomycin/penicillin	Gibco	Cat#15140122
GlutaMAX	Gibco	Cat#35050061
Tryptose Phosphate Broth	Gibco	Cat#18050039
Hanks balanced saline solution	Sigma-Aldrich	Cat#H9394
Collagenase P	Roche	Cat#11 213 857 001
Dispase II	Roche	Cat#04942078001
DNase I	Roche	Cat#10104159001
PerfeCTa SYBR® Green FastMix	Quantabio	Cat#95072-250
Fetal bovine serum	Sigma Aldrich	Cat#F9665-500ML
RNAlater solution	Sigma Aldrich	Cat#R0901-500ML
TRIzol	Invitrogen	Cat#15596026
2X MEM (Temin’s modification)	Gibco	Cat#11935046
Dulbecco’s Modified Eagle Medium (DMEM)	Gibco	Cat#21969-035

**Critical commercial assays**		

Tumor-Associated Fibroblast Isolation Kit, mouse	Miltenyi Biotec	Cat#130-116-474
QIAquick PCR Purification Kit	Qiagen	Cat#28106
High-Capacity RNA-to-cDNA Kit	Applied Biosystems	Cat#4387406
PureLink RNA Mini Extraction Kit	Invitrogen	Cat#12183018A
PureLink™ RNA Micro Scale Kit	Invitrogen	Cat#12183016
Cytofix/Cytoperm Fixation/Permeabilization Kit	BD	Cat#554714
Zymo Quick-RNA Microprep Kit	Zymo Research	Cat#R1050

**Experimental models: Cell lines**		

Baby Hamster Kidney-21	Derived from Lefteri et al.^5^	RRID: CVCL_1914
Vero cells	Derived from Lefteri et al.^5^	RRID: CVCL_0059

**Experimental models: Organisms/strains**		

B6(Cg)-*Ifnar1*tm1.2Ees/J mice Strain #:028288 (Mus musculus; Ifnar1^−/−^, homozygous; male and female; 6–10 weeks of age)	Jackson Laboratory	RRID: IMSR_JAX:028288
Phlebotomus perniciosus (Murcia, Spain; laboratory colony)	P. Volf, Charles University, Czech Republic	N/A
Lutzomyia longipalpis (laboratory colony)	M.E. Rogers, LSHTM, UK	N/A

**Oligonucleotides**		

See Table 1 for all sequences	N/A	N/A

**Software and algorithms**		

CytExpert software; CytoFLEX Platform	Beckman Coulter	N/A
GraphPad Prism	GraphPad Software	RRID: SCR_002798

**Other**		

MS Columns	Miltenyi Biotec	Cat#130-042-201
LD Columns	Miltenyi Biotec	Cat#130-042-901
QIAshredder column	Qiagen	Cat#79656
Anti-Rat/Hamster Ig κ/Negative control compensation beads	BD	Cat#552845
Compensation Beads	Biolegend	Cat# 424601
Anti-Mouse CD16/CD32 (Mouse BD Fc Block) (1:50)	BD Pharmingen	Cat#553141
Stainless-steel beads 7mm	Qiagen	Cat#69990
Hamilton 75 RN syringe (5 μl)	VWR	548-2272
Custom 33G point 4 style microneedles	VWR	HAMI7803-05
Isoflurane vaporiser and induction chamber	VetTech Solutions Ltd	Cat# AN00110; Cat#AN003A
Isoflurane (IsoFlo)	Henry Schein	N/A
Sedator (medetomidine)	Dechra	N/A
Ketavet (ketamine)	Zoetis	N/A
Revertor (atipamezole)	Dechra	N/A
TissueLyser II	Qiagen	Cat#85600
Thermomixer F2.0 (with 1.5 ml tube block)	Eppendorf	EP5387000030
CytoFLEX flow cytometer	Beckman Coulter	N/A
QuantStudio 7 Flex Real-Time PCR System	Applied Biosystems	N/A
MACS MultiStand	Miltenyi Biotec	Cat#130-042-303
MiniMACS Separator	Miltenyi Biotec	Cat#130-042-102
Agilent 2100 Bioanalyzer	Agilent	Cat#G2939BA
RNA Pico chip kit (Bioanalyzer)	Agilent	Cat#5067-1513
Tapestation 2400 system	Agilent	Cat#G2991BA
Spectrophotometer (NanoDrop or equivalent)	Thermo Fisher	N/A
70μm cell strainer	Corning	CLS352340
96-well round-bottom plates	Thermo Scientific	163320
T75 tissue culture flasks	Corning	CLS430639
12-well tissue culture plates	Corning	CLS3513
1.5 ml microcentrifuge tubes (sterile)	Starlab	E1415-1510
Cryovials (sterile)	STarlab	E3110-6122
Centrifuge (refrigerated)	Thermofisher	Sorvall SL8
Zeiss Axioscan Z1 slide scanner	Zeiss	N/A

**Table 1:** List of primers, primer orientation, sequences, product size and NCBI reference

Primer pair	Target gene	Orientation	Sequence	Product size (bp)	NCBI reference
18S	18S rRNA	Forward	gactcaacacgggaaacctc	124	NR_003278. 1
		Reverse	taaccagacaaatcgctccac		
18S Standard	18S rRNA	Forward	cgtagttccgaccataaacga	443	NR_003278. 1
		Reverse	acatctaagggcatcacagacc		
TOSV NSs	NSs	Forward	gaaccagactttacgagccaac	102	X53794.1
		Reverse	gccacctgagagcagacaa		
TOSV NSs Standard	NSs	Forward	ggttcaggccacaagaggt	519	X53794.1
		Reverse	agcagtcaatccgtgctttt		
TOSV N	N	Forward	tcatggctcttgggtaggtt	118	X53794.1
		Reverse	gtgtcagccgcatttgttc		
TOSV N Standard	N	Forward	cgatacttctcagcactcctcac	540	X53794.1
		Reverse	caggggaaacaagccaga		

## Materials and equipment

**Table:** Digestion solution

Reagent	Final concentration	Amount (per sample)
Hank’s Balanced Salt Solution (HBSS)	1x	900 μL
Collagenase P	1 mg/mL	100 μL
Dispase II	0.5 mg/mL	50 μL
DNase I	0.1 mg/mL	8 μL
**Total volume**	–	**1.058 mL**

Keep the solution on ice and always prepare it fresh.

**CRITICAL:** Prepare solution using fresh enzymes and avoid freeze-thawing reagents.

**Table:** FACS buffer

Reagent	Final concentration	Amount (100 mL)
PBS without Ca^2+^/Mg^2+^ (pH 7.2)	1x	Up to 100 mL
Fetal Calf Serum	0.5%	0.5 mL
EDTA (0.5 M stock)	2 mM	0.4 mL
**Total volume**	–	**100 mL**

Store at 4°C for up to 2 months.

**CRITICAL:** Keep buffer cold and use pre-cooled solutions during staining to maintain cell viability.

**Table:** MACS buffer

Reagent	Final concentration	Amount (100 mL)
PBS without Ca^2+^/Mg^2+^ (pH 7.2)	1x	Up to 100 mL
Fetal Calf Serum	0.5%	0.5 mL
EDTA (0.5 M stock)	2 mM	0.4 mL
**Total volume**	–	**100 mL**

Store at 4°C for up to 2 months.

**CRITICAL:** After mixing, sterilize the buffer by filtration through a 0.22 μm filter.

**Table:** Flow cytometry staining panel for detection of neutrophil, macrophages

Antibody	Fluorochrome	Vendor	Clone	Marker for	Final dilution
CD45	FITC	Biolegend	30-F11	common leukocyte antigen	1:200
CD11b	APC	Biolegend	M1/70	monocytes, macrophages	1:200
MERTK (Mer)	APC-Cy7	Biolegend	2B10C42	macrophages	1:200
Ly-6G	Brilliant Violet 421	Biolegend	1A8	neutrophils	1:200
TOSV	mCherry	N/A	N/A	infected TOSV cells	N/A
Live/Dead dye	Ultraviolet	Biolegend	N/A	cell viability	1:50

**Table:** Flow cytometry staining panel for detection of monocytes, dendritic cells

Antibody	Fluorochrome	Vendor	Clone	Marker for	Final dilution
CD45	FITC	Biolegend	30-F11	common leukocyte antigen	1:200
Ly-6C	PE	Biolegend	HK1.4	monocytes	1:200
MHC-II	APC	Biolegend	M5/114.15.2	antigen-presenting cells	1:200
CD11c	PE-Cy7	Biolegend	N418	dendritic cells	1:200
TOSV	mCherry	N/A	N/A	infected TOSV cells	N/A
Dead/Live	Ultraviolet	Biolegend	N/A	cell viability	1:50

**Table:** Flow cytometry staining panel for detection of skin stromal cells

Antibody	Fluorochrome	Vendor	Clone	Marker for	Final dilution
CD45	FITC	Biolegend	30-F11	common leukocyte antigen	1:200
Vimentin	APC	Biotechne	280618	mesenchymal cells, including fibroblasts	1:200
CD31	PE	Biolegend	390	pan-endothelial cells	1:200
CD326 (Ep-CAM)	APC-Cy7	Biolegend	G8.8	epithelial cells, including keratinocytes	1:200
TOSV	mCherry	N/A	N/A	infected TOSV cells	N/A
Dead/Live	Ultraviolet	Biolegend	N/A	cell viability	1:50

**Table:** Flow cytometry staining panel for detection of fibroblasts subpopulations

Antibody	Fluorochrome	Vendor	Clone	Marker for	Final dilution
CD45	FITC	Biolegend	30-F11	common leukocyte antigen	1:200
CD31	FITC	Biolegend	390	endothelial cells	1:200
CD326 (Ep-CAM)	FITC	Biolegend	G8.8	epithelial cells	1:200
CD90.2 (Thy1.2)	Pacific Blue	Biolegend	30-H12	fibroblasts	1:200
Podoplanin	Alexa Fluor 647	Biolegend	PMab-1	activated fibroblasts	1:200
CD140a (PDGFR-α)	PE-Cy7	Biolegend	APA5	activated fibroblasts	1:200
Ly-6A/E (Sca-1)	PE	Biolegend	W18174A	pluripotent mesenchymal	1:200
TOSV	mCherry	N/A	N/A	infected TOSV cells	N/A
Dead/Live	Ultraviolet	Biolegend	N/A	cell viability	1:50

## Step-by-step method details

### Generation of Toscana virus (TOSV) working stocks

**Timing: ∼4–5 days**

This step describes the amplification of TOSV (strain 1812)^4^ in Baby Hamster Kidney-21 (BHK-21) cells and preparation of clarified, titrated working stocks for *in vivo* infection. Proper stock preparation and aliquoting are essential to maintain viral infectivity and ensure reproducible inoculation across experiments.

1. Seed BHK-21 cells in a T75 to reach 80–90% confluency the following day.
2. Remove culture medium and inoculate cells with TOSV seed stock diluted in phosphate-buffered saline containing 0.75% (w/v) bovine serum albumin (PBSA) at recommended multiplicity of infection (MOI) of 0.5.
  a. Calculate the MOI based on the cell density at the time of infection.
  b. Ensure that the inoculum volume evenly covers the cell monolayer in the T75 flask.

***Note:*** If seed stock availability is limited, a lower MOI (e.g., 0.1) may also be used.

3. Incubate for 1 h at 37°C to allow viral adsorption.

**CRITICAL:** Gently swirl flask every 20 min during the incubation to ensure uniform infection of the monolayer.

4. After adsorption, add 10 mL of complete DMEM to each T75 flask.
  a. Return cells to the incubator.

***Note:*** Complete DMEM is Dulbecco’s Modified Eagle Medium (DMEM) supplemented with 5% fetal calf serum (FCS), 1% tryptose phosphate broth (TPB), 1% GlutaMAX and 1% penicillin/streptomycin.

5. Monitor daily for cytopathic effect (CPE), which in BHK-21 cells is characterized by cell rounding, increased refractility, detachment from the monolayer and loss of normal cell morphology. CPE typically develops within 3-4 days.
6. Once 70–80% of monolayer displays CPE, collect the culture supernatant (including detached cells).
7. Clarify by centrifugation (1000 *× g*, 10 min, 4°C) to remove debris.
8. Aliquot clarified supernatant into small volumes, e.g., 250 μL, in sterile cryovials.
9. Store at −80°C (short-term storage) or liquid nitrogen (long-term storage).

**Pause point:** Titrated virus stocks can be stored at −80°C, or in liquid nitrogen for long-term storage, until required.

**CRITICAL:** Prepare small-volume aliquots to avoid repeated freeze-thaw cycles, which significantly reduce viral infectivity and introduce variability between experiments.

### Plaque assay-based titration of TOSV stocks

**Timing: ∼5–7 days**

This step quantifies infectious TOSV in viral stocks or mouse serum samples using a plaque assay on BHK-21 cells. Accurate titration is essential to define the inoculum dose for reproducible *in vivo* infection experiments.

10. Seed BHK-21 cells in 12 well plates to reach 80% confluency the following day.
11. Prepare 10-fold serial dilutions of TOSV in 0.75% PBSA (typically 10^−1^ to 10^−8^), ensuring sufficient volume, e.g., 500 μL, for at least duplicate wells per dilution.

**CRITICAL:** Keep all virus dilutions on ice and use fresh pipette tips at each dilution step to prevent cross-contamination.

12. Remove culture medium and infect cells with 200 μL of each dilution.
13. Incubate for 1 h at 37°C to allow adsorption.

**CRITICAL:** Gently swirl plate every 20 min to ensure even viral distribution across the monolayer.

14. Overlay cells with 2 mL of a 1:1 mixture of 2xMEM and 1.2% Avicel.
15. Incubate the plates undisturbed for 3 days at 37°C.

**CRITICAL:** Do not move plates during incubation, as disturbance can disrupt plaque formation.

16. Remove overlay and fix cells with 10% PFA (30 min, 20–25°C).
17. Stain cells with 1% crystal violet (30 min, 20°C–25°C).
18. Rinse the wells gently with tap water to remove excess dye and allow the plate to air-dry.
19. Count plaques to determine the titer in plaque-forming units (PFU) (Figures 1A and 1B).

**Figure 1:**
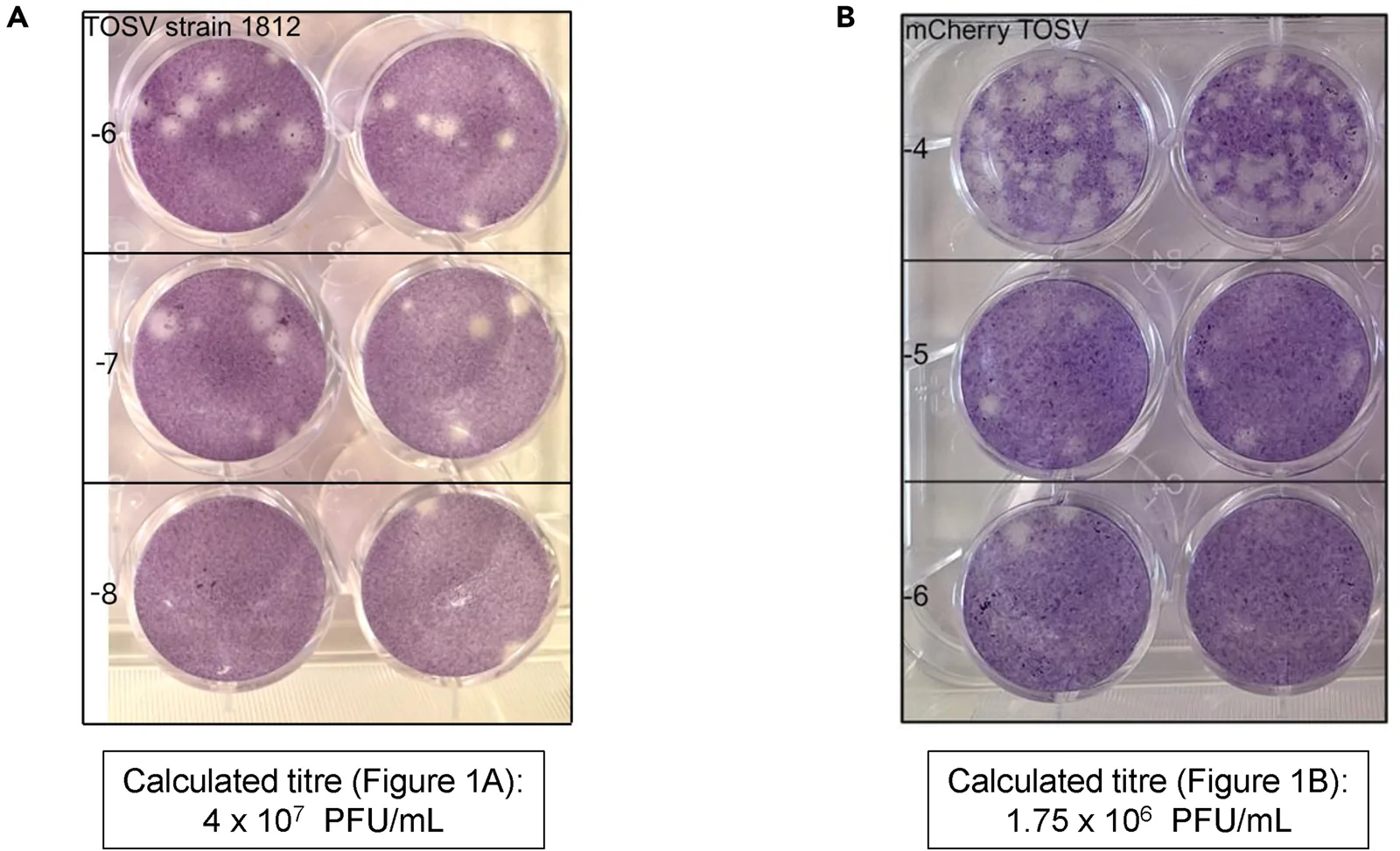
Representative plaque assay results and calculated viral titres Representative plaque assay results for TOSV strain 1812 (A) and TOSV-mCherry (B) following serial dilution on BHK-21 cells. Viral titres were calculated using the formula: PFU/mL = (average plaque count x dilution factor)/volume plated (mL). Calculated titres were 4 x 10^7^ PFU/mL for TOSV strain 1812 and 1.75 x 10^6^ PFU/mL for TOSV-mCherry.

***Note:*** If the assay is unsuccessful, see troubleshooting 1.

### Preparation of TOSV inoculum for subcutaneous foot skin infection

**Timing: ∼30 min**

This step describes preparation of TOSV inoculum for subcutaneous infection of *Ifnar1*^−/−^ mice, either alone or mixed with SGE. Accurate dilution and handling of small volumes are essential for consistent delivery of defined infectious doses.

20. Retrieve a frozen aliquot of titrated TOSV working stock and thaw rapidly. Keep on ice thereafter.
21. Dilute the virus in sterile 0.75% PBSA to achieve the desired PFU per injection volume.
22. For SGE experimental groups, prepare a 1:1 mixture of diluted TOSV and SGE and keep on ice until injection.
  a. Use SGE at 1 gland equivalent per μL, giving a final concentration of 0.5 gland equivalents per μL in the inoculum and delivering 1 gland equivalent per mouse in a 2 μL injection unless otherwise stated.
  b. Dilute the virus so that the intended dose (e.g., 10^5^ PFU) is delivered in the final 2 μL after 1:1 mixing.
23. Dilute virus-only controls 1:1 in 0.75% PBSA so that both groups of mice receive the same dose in the same volume.
24. Transport inoculum to the animal facility on ice.

**CRITICAL:** This protocol uses custom-made 33G (point 4 style) microneedles and a 5μL Hamilton 75 RN glass syringe for subcutaneous inoculation delivery into the upper foot skin. Prepare the virus inoculum in sterile 1.5 mL microcentrifuge tubes that allow easier aspiration of the mixture into the syringe. To ensure reproducible downstream viral RNA quantification and cell recovery, collect the same defined anatomical region from all animals during tissue collection (see Step 44).

**CRITICAL:** Prepare fresh virus-SGE mixtures immediately before inoculation. Avoid repeated freeze-thaw cycles of SGE aliquots.

***Note:*** Once the viral stock titers are defined, adjust dilution to achieve the desired infectious dose per mouse. In this study, 10^5^ PFU per injection was optimal for TOSV strain 1812 in *Ifnar1−/−* mice. The effective titre may vary depending on viral strain, route of administration, injection site and mouse genotype; optimization is recommended.

***Note:****Phlebotomus perniciosus* (Murcia, Spain) SGE was kindly provided by Petr Volf (Charles University, Czech Republic) in aliquots of 100 glands/100μL or 10 glands/10μL of 0.9% NaCl. *Lutzomyia longipalpis* SGE were kindly provided by Dr. Matthew Rogers (London School of Hygiene and Tropical Medicine, United Kingdom). Four-day-old, non-blood-fed female sand flies were immobilized on ice prior to salivary gland dissection. Under a stereomicroscope, salivary glands were isolated by removing the head and dissecting the glands into phosphate-buffered saline. Glands were disrupted either by 10 s sonication or by two freeze-thaw cycles, followed by centrifugation at 10,000 *× g* for 2 min to collect the supernatant.^6^^,^^7^^,^^8^ A detailed video demonstration of this procedure is available, sand fly salivary glands dissection. The term “SGE” refers to salivary gland extract obtained following gland dissection and disruption, “saliva” to refer to the material naturally deposited during sand fly feeding, and “bite” to indicate the live feeding condition.

***Note:*** SGE quality control. For *working concentration, e*xpress doses as salivary gland equivalents in the final inoculum, accounting for the 1:1 dilution with virus, and record the injected volume for each experiment. For *Inter-batch SGE consistency, q*uantify total protein in each SGE batch using a NanoDrop spectrophotometer (absorbance at 280 nm) and express content as μg protein per gland equivalent. This provides a rapid relative measure for comparing batches rather than absolute quantification; a colorimetric assay (e.g. BCA or Bradford) against a BSA standard curve can be used where absolute values are required. Batches differing by more than ∼20% should not be combined within a study. Comparing batch protein profiles by SDS-PAGE can provide an additional qualitative identity check. For *storage stability,* store SGE at −70°C in single-use aliquots sized for one experiment, for up to 24 months. Thaw once, keep on ice and do not refreeze; no more than one freeze-thaw cycle is recommended. Where possible, generate single large batches so that all animals within an experiment receive SGE from the same preparation.

*Species.* SGE from *P. perniciosus* and *Lu. longipalpis* are not interchangeable: salivary protein composition differs between species and the magnitude of enhancement differs accordingly. Use a single species within an experiment and state the species and dose in figure legends.

### Optional: Controlled sand fly blood-feeding on upper foot skin prior to TOSV inoculation

**Timing: ∼30 min per mouse**

This step describes controlled exposure of *Ifnar1*^−/−^ mice to female sand flies to allow blood feeding on the upper foot skin prior to virus infection. This can be used to investigate vector-mediated transmission and assess the saliva-driven modulation of TOSV infection.

25. Prepare female sand flies (∼1 week old).
  a. Starve the flies by removing sugar 16 to 18 h prior to feeding to promote blood meal uptake.
26. Prepare a custom ventilated feeding chamber that protects the mouse body parts whilst leaving the upper foot skin exposed (Figure 2A).

**Figure 2:**
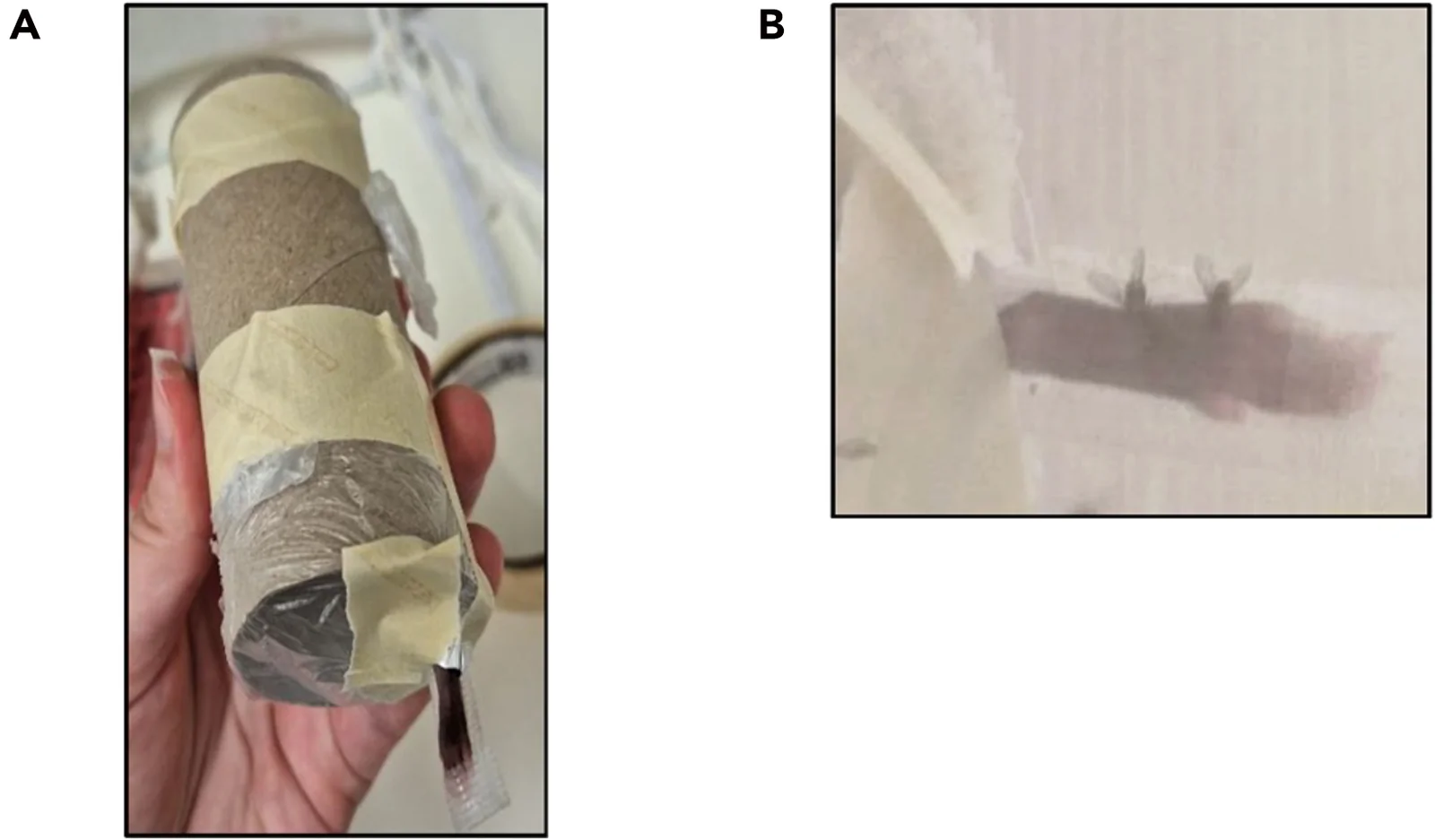
Experimental setup for controlled sand fly blood-feeding on mouse upper foot skin (A) A specially designed box that ensures adequate airflow for mice during blood feeding. Note one end of the box, not shown, is open to allow airflow. (B) Two female sand flies were feeding on the upper foot skin of a mouse .
27. Anesthetize mice with 0.1 mL/10 g of Sedator®/Ketavet via intraperitoneal injection.
28. Place the mouse into the sand fly cage and allow 2–4 female sandflies per foot to blood-feed.
  a. Allow sandflies to feed until fully engorged (Figure 2B).
29. Immediately following confirmed feeding, inject the virus subcutaneously into the exposed bitten area of the dorsal foot skin.

**CRITICAL:** Ensure consistent number of bites per mouse to reduce variability.

**CRITICAL:** Cover the toes with sterile tape to prevent unintended biting of distal digits.

***Note:*** Blood feeding is restricted to a small, defined area of the upper foot skin; proximity to the bite sites is sufficient to produce local saliva-mediated effects.

***Note:*** It is recommended that male sand flies be introduced into the cages at a male-to-female ratio of 1:2 to stimulate biting behaviors in females.

***Note:*** Individual bite sites are not reliably visible at this early time point, as feeding does not leave an identifiable mark within the minutes between blood-feeding and inoculation. Reproducibility is instead achieved by restricting the area available for feeding: the ventilated chamber and taping of the toes expose only a small, defined region of dorsal upper foot skin, so all bites are confined to that region. Feeding is confirmed by engorgement of the flies (Figure 2B) rather than by inspection of the skin. The virus is injected centrally within this exposed region and is therefore delivered into sand fly-bitten skin, but is not aimed at an individual bite. Given the small size of the exposed area relative to the spread of the subcutaneous bleb, proximity within the bitten region is sufficient and exact superimposition on a single bite is neither achievable nor required.

### Subcutaneous infection of *Ifnar1*^−/−^ mice via upper foot skin inoculation

**Timing: ∼10–15 min per mouse**

This step describes inhalation anesthesia and subcutaneous inoculation of *Ifnar1*^−/−^ mice with TOSV, either alone, combined with SGE, or following sand fly exposure, into the dorsal upper foot skin (not the footpad). Precise anatomical placement and controlled anesthesia are critical to ensure reproducible local infection and downstream skin-specific analyses.

30. Ensure that all infection materials (virus inoculum, syringes, disinfectants, wipes and related tools) are fully prepared in the biosafety cabinet before anesthetizing the animals.
31. Induce anesthesia using isoflurane (4-5% for induction, ∼1.5–2 L/min oxygen flow).
32. Monitor mice closely. Once fully anesthetized (loss of righting reflex, slow and regular but deep breathing), reduce isoflurane concentration to 3% for maintenance.
33. Remove mice individually and proceed immediately to inoculation.

**CRITICAL:** This protocol uses inhalation anesthesia because it allows rapid induction and recovery, minimizing stress and enabling quick handling. However, prolonged exposure to ≥5% isoflurane can be fatal. Continuously monitor respiratory rate and reflexes throughout the procedure.

***Note:*** For experiments involving sand fly biting, mice were anesthetized separately using injectable anesthesia as described in the above section.

34. Place the anesthetized mouse in a prone position with the dorsal side facing upward.
35. Gently extend the hind limb to expose the upper foot skin, preferably left side (not the footpad).
36. Stabilize the foot without overstretching the skin.
37. Load the prepared inoculum into a 5 μL Hamilton syringe.
38. Insert the needle bevel-up at approximately 45° into the upper foot skin to achieve subcutaneous (not intradermal or intramuscular) delivery (Figure 3A).

**Figure 3:**
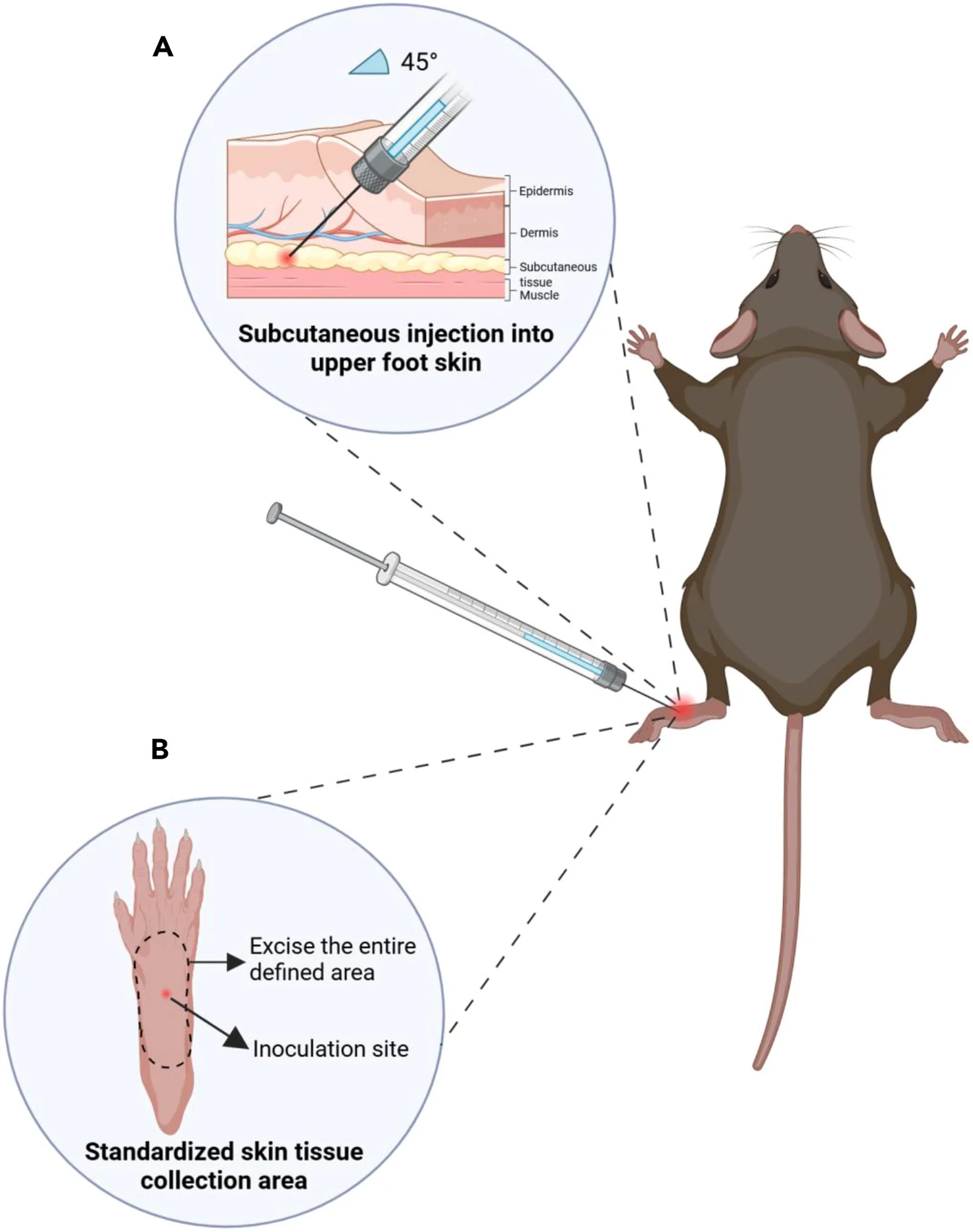
Subcutaneous inoculation and standardized skin tissue collection from the upper foot skin (A) Schematic of subcutaneous TOSV inoculation into the upper foot skin using a 45° needle angle. (B) Schematic illustrating excision of the entire upper foot skin surrounding the inoculation site for downstream analyses. Created with BioRender.com.
39. Slowly inject the defined volume (e.g., 2 μL). A small visible bleb at the injection site confirms correct placement.
40. Withdraw the needle carefully and verify absence of leakage or bleeding.
41. Confirm normal posture, mobility and respiration before returning animals to their home cage.

***Note:*** Maintaining a consistent inoculation site (e.g., left foot) across experiments minimizes sampling variability and reduces the risk of harvesting uninfected skin during downstream tissue collection.

***Note:*** If leakage occurs, see troubleshooting 2.

### Collection of skin and systemic tissues at defined time points post-infection

**Timing: ∼10–20 min per mouse (depending on number of tissues collected)**

This step describes the standardized excision of the upper foot inoculation site and optional collection of systemic tissues for downstream analyses such as flow cytometry or RNA extraction.

42. Define sampling time points according to the experimental objective.

***Note:*** In this protocol, 72 h post-infection (hpi) was used as the earliest reliable time point for consistent viral RNA detection across tissues.

43. Cull mice in accordance with institutional and governmental animal welfare regulations.
44. Place the mouse in a supine position and disinfect the skin with 70% Ethanol.
  a. Secure limbs using dissection pins and identify the dorsal foot skin inoculation site (upper surface; not the footpad).
  b. Using sterile scissors and forceps, excise the entire dorsal (upper side) foot skin surrounding the inoculation site, using consistent anatomical boundaries for all animals to ensure that the complete inoculation site is collected (Figure 3B).

**CRITICAL:** Maintain consistent excision size across animals to reduce sampling variability between experimental groups.

45. Transfer the skin sample immediately into chilled complete DMEM or RNA preservation solution depending on downstream application.

**Pause point:** Skin samples placed in RNA preservation solution can be stored at −80°C until RNA extraction. Samples intended for flow cytometry must be processed immediately and cannot be stored.

46. If required, collect additional tissues (e.g., draining lymph nodes, spleen, brain and blood) using standard techniques.
  a. Keep all samples on ice until further processing.

### Enzymatic digestion of infected upper foot skin to generate single-cell suspensions

**Timing: ∼1.5–2 h**

This step describes the enzymatic dissociation of skin tissue to obtain single-cell suspensions suitable for flow cytometry applications such as the detection of mCherry-TOSV-infected cells and downstream molecular applications (e.g., RNA extraction or RNA-seq). Proper digestion conditions are essential to preserve stromal and immune cell integrity while ensuring efficient tissue dissociation.

47. Immediately after collecting, place each skin sample into individual 1.5 mL microcentrifuge tubes containing 500 μL of ice-cold complete DMEM.
48. Keep all samples on ice until digestion.
49. Prepare one fresh digestion solution (∼1.058 mL; see materials and equipment) for each skin sample and transfer each sample into a separate 1.5 mL microcentrifuge tube containing the digestion solution.

***Note:*** Prepare sufficient digestion mix for all samples plus one extra to compensate for pipetting loss.

50. Using sterile, sharp scissors, chop the skin tissue into very small pieces directly in the digestion solution.
51. Incubate samples at 37°C for 50 min, shaking at 1100–1400 rpm (orbital shaking, not centrifugation) using a thermomixer.
  a. After incubation, immediately place tubes on ice.
  b. Add 500 μl of chilled complete DMEM to stop enzymatic activity and preserve cell viability.
52. Pass the suspension through a 70 μm cell strainer placed on a sterile 50 ml conical tube. Rinse the original tube and the cell strainer with chilled complete DMEM to maximize collection of cells.
53. Centrifuge cells at 300 *× g* for 5 min at 4°C.
  a. Carefully aspirate the supernatant.
54. Resuspend the cell pellet in an appropriate volume of complete DMEM or appropriate downstream buffer (e.g., FACS buffer or MACS buffer).

**CRITICAL:** Avoid vigorous pipetting, which can shear fragile cells.

***Note:*** If cell recovery is low, see troubleshooting 3.

### Detection of mCherry-TOSV-infected skin cells by flow cytometry

**Timing: ∼3 h (excluding acquisition and data analysis)**

This step describes the detection of TOSV-infected cells in skin-derived single-cell suspensions using a recombinant TOSV expressing the fluorescent reporter mCherry (TOSV-mCherry).^3^ This approach enables direct identification of infected cells and simultaneous phenotypic characterization using surface markers.

55. Transfer the entire skin-derived single-cell suspension into a 96 well round-bottom plate. Where cell yields allow, plate equal numbers of cells across experimental groups and record the total number stained per sample.
56. Wash cells twice with FACS buffer (PBS without Ca^2+^/Mg^2+^, 0.5% FBS, 2 mM EDTA) at 300 x *g* for 5 min at 4°C.
57. Resuspend cells in Fc blocking solution.
  a. Incubate on ice for 5 min.
58. Add antibody master mix (pre-optimized panel for stromal and immune markers).
  a. Incubate 45 min at 4°C in the dark.

***Note:*** Refer to the materials and equipment section for the antibody panels used in this study.

59. Wash cells twice with PBS (300 x *g* for 5 min at 4°C).
60. Prepare Dead/Live solution in PBS buffer [Zombie UV (Biolegend) is used in this protocol zombie-uv-fixable-viability-kit].
  a. Add Dead/Live solution to cells and incubate 10 min at 20–25°C in the dark.
  b. Wash cells twice with FACS buffer (300 x g for 5 min at 4°C).
61. Fix cells in 4% paraformaldehyde for 30 min at 20°C–25°C.
  a. Wash once and resuspend cells in FACS buffer.
  b. Store samples at 4°C in the dark until acquisition.

**Pause point:** Fixed, stained samples can be stored at 4°C in the dark for up to 48 h before acquisition.

62. Acquire samples on a flow cytometer equipped with appropriate lasers for mCherry detection (e.g., 561 nm excitation).
63. Apply compensation using appropriate single-stained controls or compensation beads prior to gating.
64. Identify infected cells based on mCherry positivity within the live, single-cell population.

**CRITICAL:** Include the following controls in each experiment: uninfected skin samples (to define background fluorescence reference), fluorescence minus one (FMO) controls, each containing all antibodies in the staining panel except the marker of interest, to enable accurate gate placement and single-stained compensation controls (e.g., compensation-beads).

***Note:*** If cell populations are poorly resolved or compensation is inaccurate, see troubleshooting 4.

***Note:*** If intracellular markers (e.g., vimentin) are included in the antibody panel, perform an additional fixation and permeabilization step prior to staining according to the manufacturer's instructions for intracellular staining buffers flow-cytometry-reagents/fixation-permeabilization-kit.

***Note:*** This workflow is optimized for genetically modified TOSV expressing mCherry. For wild-type TOSV strains, detection of infected cells would require intracellular viral antigen staining, which is not covered in this protocol.

***Note:*** In this protocol, infected cells are identified based on endogenous mCherry fluorescence, which is expressed exclusively in TOSV-infected cells. This strategy allows direct detection of infected cells in freshly isolated skin suspensions. The recombinant reporter virus TOSV ΔNSs:mCherry was generated from lineage A clinical isolate TOSV (strain 1500590) by replacing the NSs gene with the mCherry reporter gene.^3^ Note that this reporter virus lacks NSs, the principal type I IFN antagonist of phleboviruses such as TOSV, and is therefore expected to replicate inefficiently in IFN-competent hosts. Its use in this protocol is restricted to *Ifnar1*−/− mice, in which type I IFN signalling is already absent (see limitations).

***Note:*** The antibodies used in the flow cytometry staining panels target extracellular markers, apart from vimentin antibody, which is intracellular.

### Optional: Magnetic enrichment of skin fibroblasts

**Timing: ∼1.5–2 h**

This optional step enriches CD90.2^+^ fibroblasts from skin-derived single-cell suspensions to enable cell type specific downstream analyses such as viral RNA quantification. Tumour-Associated Fibroblast Isolation Kit (Miltenyi Biotec) enables negative depletion of non-fibroblast populations followed by positive selection of CD90.2^+^ fibroblasts. All named kits were used according to the manufacturer’s instructions, unless otherwise stated miltenyibiotec/tumor-associated-fibroblast-isolation-kit-mouse.

65. Prepare skin-derived single cell suspensions as described in the enzymatic digestion of infected upper foot skin to generate single-cell suspensions step.
  a. Centrifuge at 300 *× g* for 10 min at 4°C.
66. Resuspend cells in MACS buffer (PBS without Ca^2+^/Mg^2+^, 0.5% FBS, 2 mM EDTA).
67. Adjust volume to 90 μL MACS buffer per 1 x 10^7^ cells.
68. Add the Non-Tumor-Associated Fibroblast Depletion Cocktail.
  a. Mix gently and incubate for 15 min at 4°C.
  b. Wash cells by adding MACS buffer and centrifuge at 300 *× g* for 10 min at 4°C.
  c. Resuspend pellet in the buffer.
69. Apply the cell suspension onto an LD column placed in a magnetic separator.
70. Collect the flow-through fraction, which contains enriched fibroblasts (non-target cells retained in column).
71. Label fibroblasts by adding CD90.2 (Thy1.2) MicroBeads (20 μL per 1 x 10^7^ cells).
  a. Incubate for 15 min at 4°C.
  b. Wash cells with MACS buffer and centrifuge at 300 *× g* for 10 min at 4°C.
  c. Resuspend pellet in the buffer.
72. Apply the cell suspension onto an MS column placed in the magnetic separator.
73. Remove the column from the magnet and flush to elute CD90.2^+^ fibroblasts.
74. Count isolated fibroblasts and assess viability.
75. Validate fibroblast enrichment by flow cytometry using fibroblast markers (e.g., CD90.2).

**CRITICAL:** Always verify fibroblast purity before downstream analyses.

***Note:*** If purity or viability is compromised, see troubleshooting 5.

### Optional: RNA extraction from skin tissue or skin-derived cells and downstream viral RNA analysis

**Timing: ∼2–4 h (RNA extraction) + downstream application-dependent**

This step describes RNA extraction from whole skin tissue collected at the skin inoculation site or from skin-derived cell populations (bulk single-cell suspensions or MACS-enriched fibroblasts) generated in this protocol, followed by downstream applications such as RT-qPCR or RNA sequencing. In this study, RNA extraction was performed using: Zymo Quick-RNA Microprep Kit (Zymo Research) zymoresearch/quick-rna-microprep-kit, Invitrogen PureLink RNA Mini Kit thermofisher/PureLink RNA Mini Kit and Invitrogen PureLink Micro Kit thermofisher/PureLink RNA Micro Scale Kit. All named kits were used according to the manufacturer’s instructions unless otherwise specified.

76. Prepare one of the following sample types:
  a. Upper foot skin tissue collected at defined time points,
  b. Bulk skin-derived single-cell suspensions,
  c. MACS-enriched fibroblasts.
77. For skin-derived cell suspensions and MACS-enriched fibroblasts:
  a. Centrifuge cells at 300 *× g* for 5 min at 4°C to pellet cells.
  b. Carefully discard the supernatant, leaving cell pellets.
  c. Lyse cell pellets directly in lysis buffer supplemented with β-mercaptoethanol according to kit instructions.
  d. Homogenize lysates using a QIAshredder column or by vigorous pipetting if the column step is omitted.
78. For skin tissue samples:
  a. Process tissue immediately for RNA extraction or place tissue in RNA stabilizer solution and store at −80°C until extraction.
  b. If tissue has been stored in RNA stabilizer solution, remove the tissue from the solution.
  c. Add 1 mL TRIzol reagent (Invitrogen, UK) and two 7 mm stainless-steel beads to skin tissue samples.
  d. Homogenize using a TissueLyser (50 Hz, 10 min).

**CRITICAL:** Skin tissue is fibrous and difficult to disrupt. Incomplete homogenization significantly reduces RNA yield and quality.

79. For lysates from skin tissue and cell pellets, proceed with column-based RNA purification following kit instructions.

***Note:*** Include on-column DNase digestion when RNA is intended for RT-qPCR or RNA sequencing.

80. Measure RNA concentration and purity using a spectrophotometer.

**CRITICAL:** Low-input fibroblast enriched samples should be processed using the RNA purification kits optimized for small cell numbers to maximize RNA recovery (e.g., Zymo Quick-RNA Microprep Kit). Using this approach, fibroblast-enriched samples intended for RNA sequencing yielded RNA concentrations ranging from approximately 330 to 2,900 pg/μL.

**CRITICAL:** No carrier RNA or transfer RNA was added during extraction when samples were intended for RNA-seq to avoid interference with downstream library preparation and transcriptomic analyses.

81. Use extracted RNA immediately or store at −80°C until analysis.

**Pause point:** Extracted RNA can be stored at −80°C until analysis.

***Note:*** If RNA quality is poor, see troubleshooting 6.

## Expected outcomes

This protocol enables reproducible establishment of a murine TOSV skin infection model in *Ifnar1*^−/−^ mice, suitable for downstream cellular and molecular analyses. Following s.c. inoculation, viral RNA is reliably detectable at the skin inoculation site at defined post-infection time points (e.g., 72 hpi). Inclusion of sand fly SGE, and inoculation into sand fly-bitten skin, both enhance viral RNA load at the skin inoculation site (Figures 4A and 4B) and increase viral dissemination and clinical signs. Higher quantities of viral RNA are detected in peripheral tissues (e.g., spleen) than in mice infected with TOSV alone (Figures 4C and 4D). The magnitude of enhancement is graded, being greater with increasing SGE dose^1^ and greatest when virus is inoculated into bitten skin.^1^

**Figure 4:**
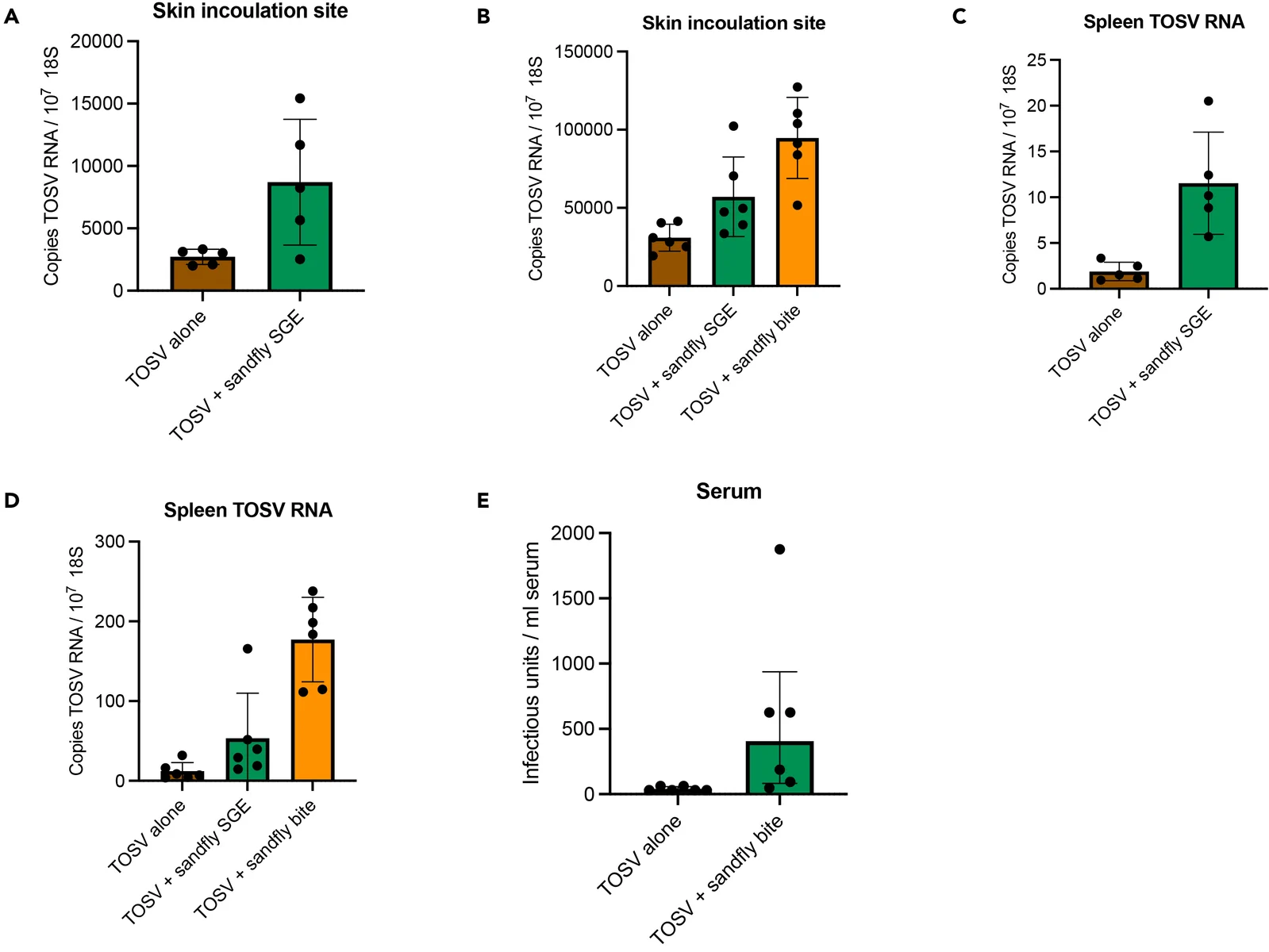
Sand fly SGE and biting enhance susceptibility to TOSV infection in mice (A and C) *Ifnar1*^−/−^ mice (n = 5) were infected with 10^5^ PFU TOSV with or without *P. perniciosus* sand fly salivary gland extract (SGE). (B and D) *Ifnar1*^−/−^ mice (n = 6) were infected with 10^5^ PFU TOSV alone, mixed with *1 Lu. Longipalpis* sand fly SGE or injected into *Lu. Longipalpis* bitten skin. (E) Serum from mice infected with 10^5^ PFU TOSV alone or injected into *Lu. longipalpis*-bitten skin. Virus RNA was quantified by qPCR and infectious units in serum defined by plaque assay. Plots show the median value ± interquartile range. Data adapted from Keskek Turk et al.^1^ under CC BY 4.0.

Infectious virus in serum is a less sensitive endpoint. At 72 hpi, infectious virus was readily detected in serum from mice inoculated into sand fly-bitten skin, whereas none of the virus-only controls had detectable viraemia (Figure 4E). Occasional viraemia in mice given SGE has been reported previously.^1^ Users should therefore expect viral RNA at the inoculation site, and in spleen, to be the more robust readouts, and should not rely on serum plaque assay alone to detect an effect of vector-derived factors at this time point.

Enzymatic digestion of infected skin tissue generates viable single-cell suspensions compatible with flow cytometry (Figures 5A and 5B). When recombinant TOSV expressing mCherry is used, infected cells are directly identifiable by endogenous fluorescence and can be phenotypically characterized using leukocyte and stromal markers. Uninfected controls typically show minimal background fluorescence, enabling clear discrimination of infected populations (Figures 6A and 6B).

**Figure 5:**
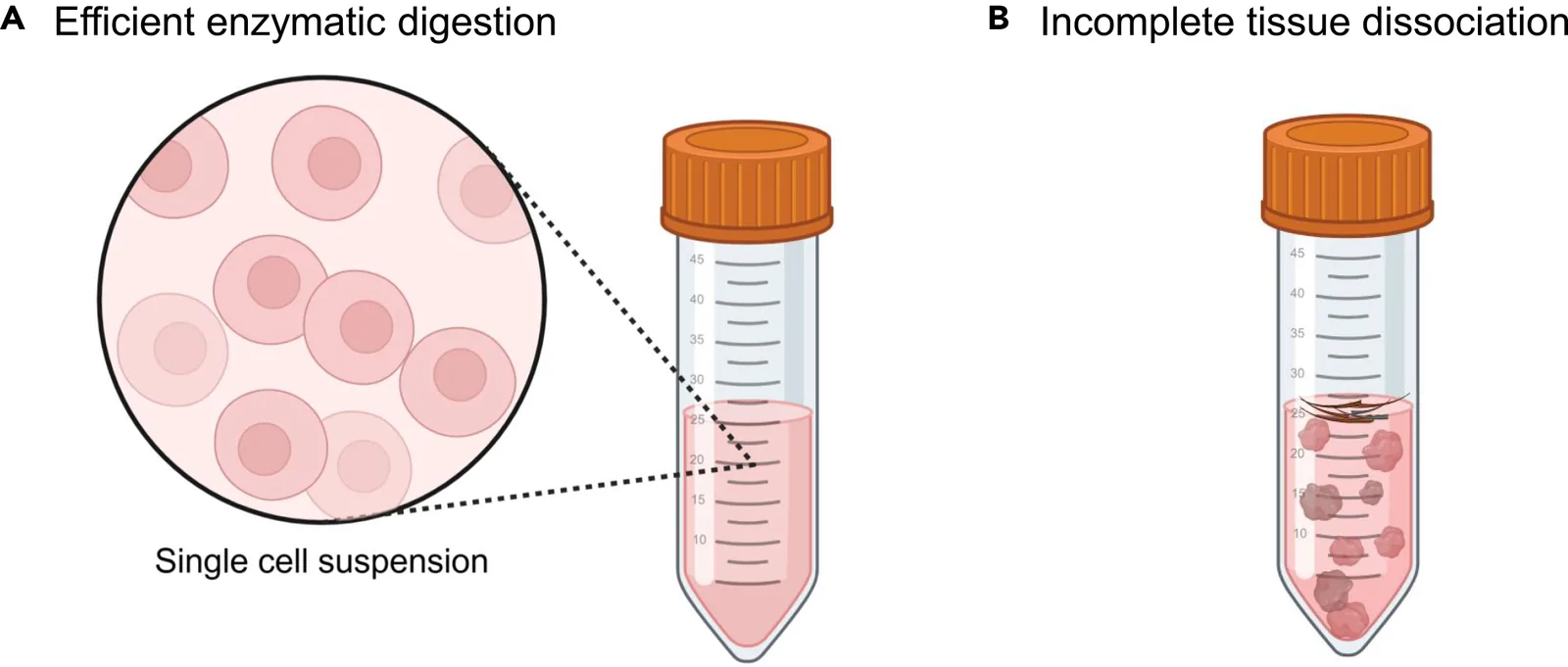
Schematic illustration of expected outcomes following skin digestion (A) Efficient digestion results in a homogeneous, cloudy single-cell suspension. (B) Incomplete digestion leaves visible tissue fragments and hair remnants, indicating insufficient enzymatic dissociation. Created with BioRender.com.

**Figure 6:**
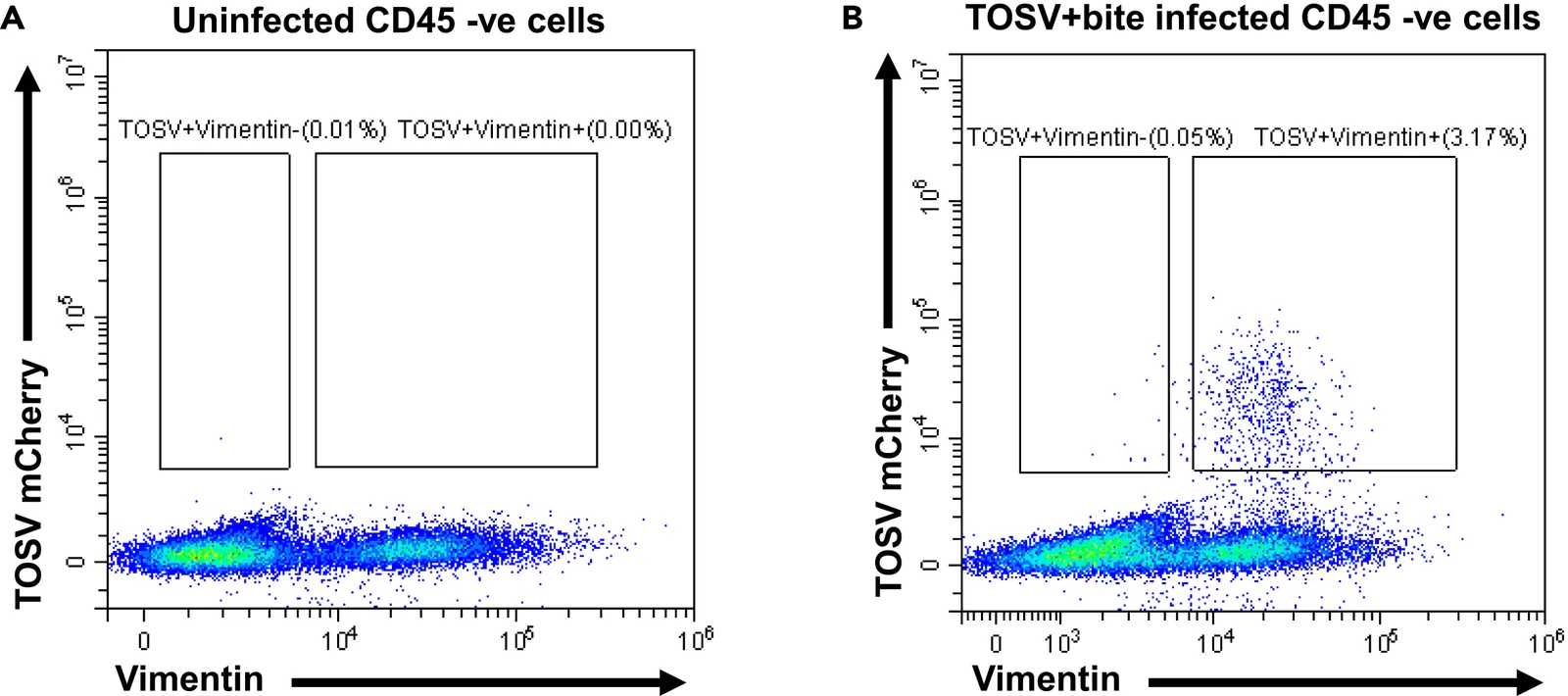
Detection of TOSV-infected cells by flow cytometry Representative flow cytometry plots showing detection of mCherry-expressing cells in skin-derived single-cell suspension following infection with recombinant TOSV-mCherry. (A) Uninfected samples show no detectable mCherry signal, whereas (B) infected samples demonstrate a distinct mCherry^+^ population. The gate defining TOSV+ cells was set using uninfected control samples lacking mCherry signal. (CD45, common leukocyte antigen; Vimentin, mesenchymal cells, including fibroblasts antigen).

Optional MACS-based enrichment yields fibroblast-enriched populations suitable for RNA extraction and downstream transcriptomic applications. RNA isolated from skin tissue or enriched-fibroblast fractions typically exhibits integrity and quantities compatible with RT-qPCR and RNA sequencing workflows (Figure 7).

**Figure 7:**
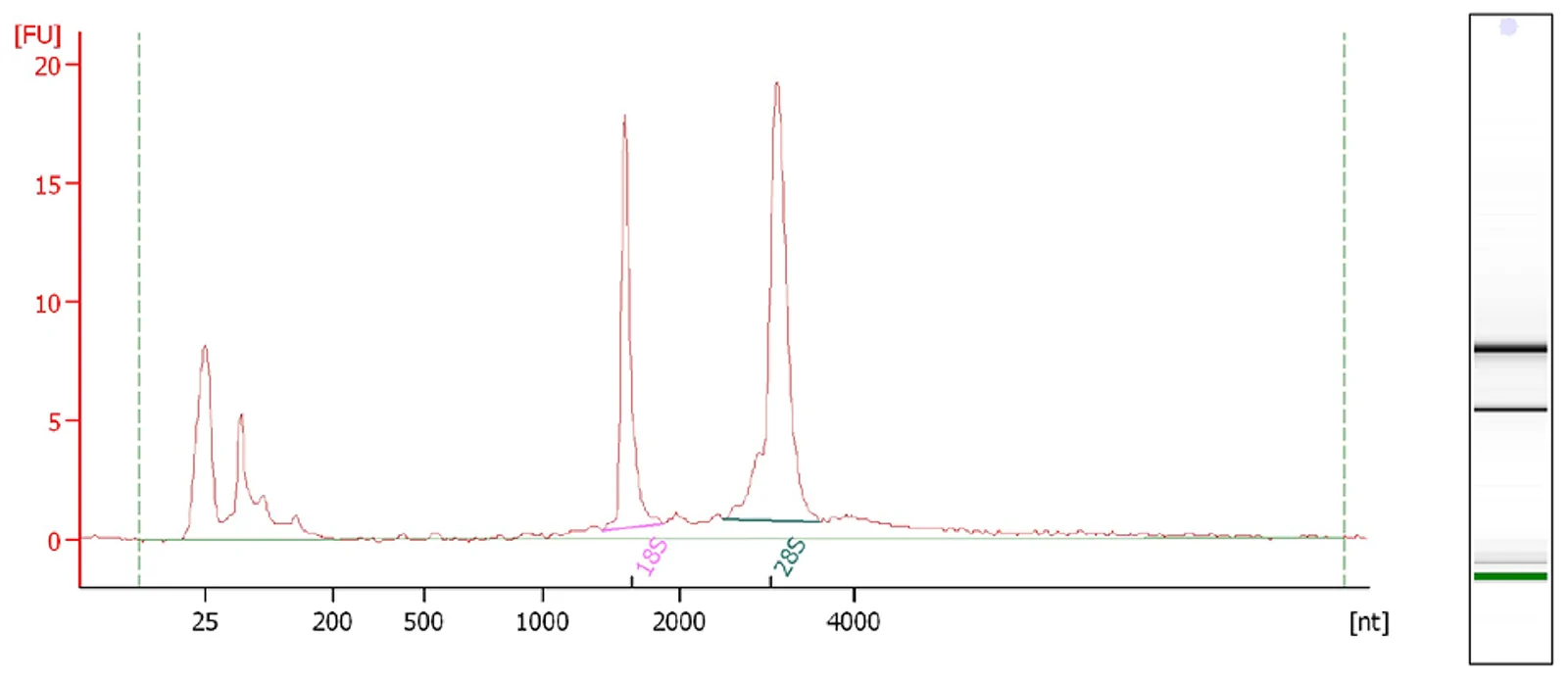
Representative RNA integrity assessment from MACS-enriched skin fibroblasts Bioanalyzer electropherogram demonstrating high-quality RNA (RIN 9.9) extracted from fibroblast-enriched skin samples. Distinct 18S and 28S rRNA peaks and minimal degradation indicate suitability for downstream applications such as RT-qPCR and RNA sequencing.

Overall, this protocol produces robust and quantifiable datasets describing TOSV infection in skin, enabling comparative analysis of experimental variables, including the presence or absence of vector-derived salivary factors.

## Quantification and statistical analysis

### The formula for titer calculation

The viral titer is calculated based on the number of plaques counted at different virus dilutions. A plaque is defined as a clear zone in the cell monolayer and the number of plaques is used to determine the infectious virus titer, expressed as plaque-forming units per milliliter (PFU/ml).

Count plaques and calculate the viral titer using the following equation:

$$\frac{PFU}{ml}=\frac{\text{Average}\;\text{number}\;\text{of}\;\text{plaques}}{\text{Inoculation}\;\text{volume}\;\left(\text{ml}\right)}x\;Dilution\;factor$$

For example, in Figure 1A, we count an average of 8 plaques from two replicate wells (10,6) at the 10^−6^ dilution using 200 μL (0.2 mL) of inoculum per well. Thus, the titer was calculated as: (8 x 10^6^)/0.2 = 4 x 10^7^ PFU/mL.

## Limitations

This protocol combines controlled subcutaneous delivery of TOSV with vector salivary exposure, achieved either by SGE or live sand fly biting, to study early virus-vector-host interactions in the skin. While this approach improves experimental reproducibility, it does not fully replicate natural transmission by infected sand flies. In the sand fly biting model, virus is administered by needle to standardize virus titer. Although biting is incorporated, the exact volume and composition of saliva delivered during feeding cannot be precisely controlled. To reduce variability, a defined number of sand flies and a fixed feeding duration are used; however, natural variations among flies remain.

Furthermore, SGE was used, because of the technical challenges associated with collecting saliva directly from sand flies. However, SGE represents a homogenized salivary gland preparation rather than naturally secreted saliva and therefore may not fully reflect the composition or dynamics of saliva released during natural feeding. In addition, some bioactive salivary components may be altered or lost during gland disruption and centrifugation.

The use of *Ifnar1*^−/−^ mice is required to establish stable infection in this model but may limit direct extrapolation to immunocompetent settings, particularly for immune responses involving type 1 interferon signaling.

Downstream applications, including fibroblast enrichment, rely on low-input samples and may result in variable RNA yield and quality.

A further consideration is that infected cells are identified using a recombinant reporter virus, TOSV ΔNSs:mCherry, in which the non-structural protein NSs has been replaced by the mCherry reporter gene. NSs is the principal type I IFN antagonist of phleboviruses, including TOSV,^9^ acting to suppress IFN- β induction, and NSs-deletant TOSV is attenuated in, and can only be propagated efficiently in, cells that are unable to mount or respond to a type I IFN response. Deletion of NSs may therefore alter viral pathogenesis relative to wild-type virus, and this should be taken into account when interpreting data obtained with the reporter virus. In the model described here this consideration is mitigated, because all infections are performed in *Ifnar1*^−/−^ mice in which type I interferon signalling is already absent and the dominant function of NSs is consequently largely redundant. For the same reason, we do not recommend use of TOSV ΔNSs:mCherry in immunocompetent wild-type mice, in which inefficient replication would be anticipated; in that setting, wild-type TOSV combined with intracellular viral antigen staining is the appropriate approach. Nonetheless, in this protocol all quantitative measurements of viral load, dissemination and clinical outcome are performed with wild-type TOSV (strain 1812), and TOSV ΔNSs:mCherry is used solely to identify and phenotype infected cells. Users should match inoculum by plaque assay titre, avoid direct comparison of datasets generated with the two viruses, and, where possible, corroborate key findings obtained with the reporter virus in parallel wild-type virus infections.

Finally, successful implementation of this protocol requires access to specialized resources, including sand fly colonies, biosafety facilities for arbovirus handling and flow cytometry instrumentation, which may limit its broader applicability.

## Troubleshooting

### Problem 1

Irregular staining and poorly defined plaques in plaque assay readout (related to Steps 10–19) (Figure 8).

**Figure 8:**
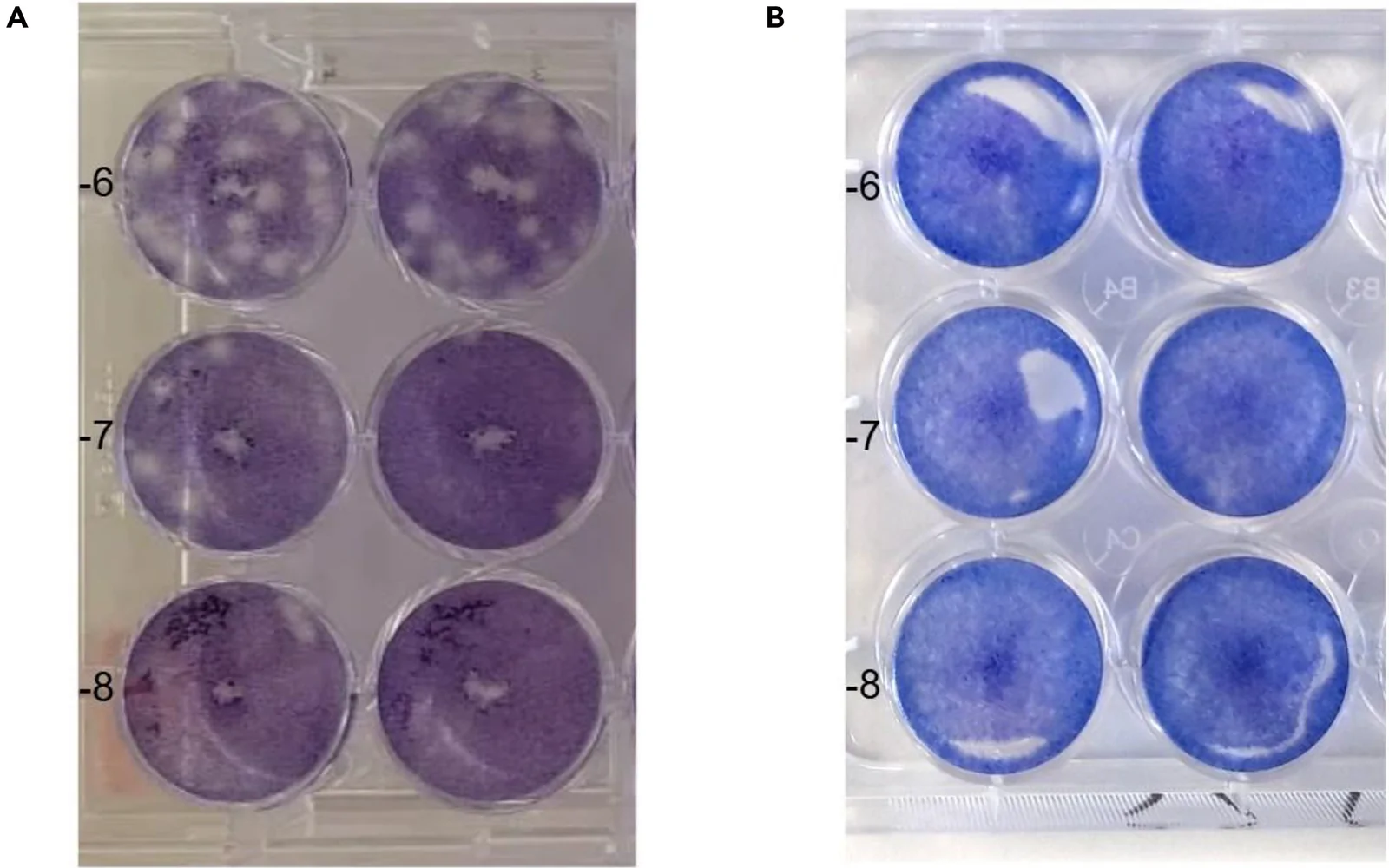
Example of unsuccessful plaque assay staining (A) Patchy staining and poorly defined plaques, with increased dye retention at 10^−8^. Central areas of cell loss may reflect overconfluent monolayers leading to cell detachment during incubation. (B) Cell loss and irregular clearing consistent with monolayer disruption and insufficient washing.

### Potential solution

Ensure cells are evenly seeded and ∼80% confluent at infection. Aspirate and add inoculum gently along the well wall to avoid monolayer damage. Dispense 2xMEM/Avicel overlay slowly down the side of the well and avoid bubble formation. Do not move plates during incubation, as overlay displacement distorts plaque formation.

### Problem 2

Inoculum leakage from the injection site (related to Steps 37–40).

### Potential solution

Ensure the needle is inserted with the bevel side facing upwards at an approximately 45° angle into the dorsal upper foot skin (not the footpad). Inject the inoculum slowly while gently stabilizing the foot. Correct subcutaneous placement is typically indicated by the formation of a small, visible bleb. If leakage occurs, reduce the injection speed and reattempt using a fresh needle at an adjacent site.

### Problem 3

Low cell yield after skin digestion (related to Steps 47–54).

### Potential solution

Ensure skin tissue is finely minced into very small fragments prior to enzymatic digestion. Confirm that tissue pieces remain fully submerged throughout incubation. If yield remains low, extend digestion time slightly to improve enzyme access. Verify digestion enzymes are active and have not undergone repeated freeze-thaw cycles.

### Problem 4

Poor separation of cell populations during flow cytometry (related to Steps 58–64).

### Potential solution

Ensure proper compensation using single-stained controls or compensation beads. Exclude debris, doublets and dead cells through sequential gating (e.g., FSC/SSC, singlets and viability dye). Confirm that cell suspensions are free of clumps prior to acquisition. If population resolution remains poor, verify antibody concentrations and perform titrations to determine optimal staining conditions, particularly for markers that overlap with the mCherry channel.

### Problem 5

Reduced cell viability or low recovery after MACS separation (related to Steps 65–75).

### Potential solution

Ensure the MACS column does not dry out during the separation procedure. Always maintain sufficient buffer volume above the column matrix while it is placed in the magnetic field. Column drying can reduce cell viability and compromise recovery.

### Problem 6

Low RNA integrity or RNA degradation (related to Steps 76–81).

### Potential solution

Maintain RNase-free conditions throughout sample handling and extraction. Process tissues or cell pellets promptly or stabilize samples in stabilization solution (e.g., RNAlater). Avoid repeated freeze-thaw cycles of RNA samples. If degradation persists, verify reagent quality and confirm that extraction kits and buffers are within their recommended storage and usage limits.

## Resource availability

### Lead contact

Further information and requests for resources and reagents should be directed to and will be fulfilled by the lead contact, Clive S. McKimmie (clive.mckimmie@york.ac.uk).

### Technical contact

Technical questions on executing this protocol should be directed to and will be answered by the technical contact, Yonca Keskek Turk (yoncaturk@hacettepe.edu.tr).

### Materials availability

This protocol did not generate new reagents.

### Data and code availability

The published article^1^ includes all datasets generated or analyzed during this protocol. Additional information is available from the corresponding author upon request.

## Acknowledgments

We gratefully acknowledge Leeds Biomedical Services and York Biological Services for technical assistance with mouse procedures and the Leeds Faculty of Medicine & Health Flow Cytometry Facility for their support. This work was funded by a scholarship to Y.K.T. from Republic of Türkiye Ministry of National Education for a Study Abroad Postgraduate Education Scholarship (MEB1416) and a 10.13039/100009001University of York startup grant. C.S.M. and A.M.-B. were funded by the 10.13039/501100000265Medical Research Council (MR/N013840/1). The graphical abstract was created with Biorender.com.

## Author contributions

Conceptualization, C.S.M. and Y.K.T.; methodology, all authors; investigation, all authors; formal analysis, Y.K.T. and C.S.M.; visualization, Y.K.T. and C.S.M.; resources, all authors; writing – original draft, Y.K.T. and C.S.M.; writing – review and editing, all authors; supervision, C.S.M.; project administration, C.S.M.; funding acquisition, all authors.

## Declaration of interests

The authors declare no competing interests.
